# Structural and Functional Analysis of PGRP-LC Indicates Exclusive Dap-Type PGN Binding in Bumblebees

**DOI:** 10.3390/ijms21072441

**Published:** 2020-04-01

**Authors:** Yanjie Liu, Nanhui Ye, Minming Chen, Huiyue Zhao, Jiandong An

**Affiliations:** 1 Key Laboratory for Insect-Pollinator Biology of the Ministry of Agriculture and Rural Affairs, Institute of Apicultural Research, Chinese Academy of Agricultural Sciences, Beijing 100093, China; liuyanjie@caas.cn (Y.L.); zhaohuiyue1124@163.com (H.Z.); 2 College of Biological Science and Engineering, Fuzhou University, Fuzhou, Fujian 350108, China; yenanhui@fzu.edu.cn (N.Y.); chenminming11@163.com (M.C.)

**Keywords:** PGRP-LC, bumblebee, *Bombus lantschouensis*, innate immune, Dap-type PGNs

## Abstract

Peptidoglycan recognition proteins (PGRPs) play an important role in the defense against invading microbes via the recognition of the immunogenic substance peptidoglycan (PGN). Bees possess fewer PGRPs than *Drosophila melanogaster* and *Anopheles gambiae* but retain two important immune pathways, the Toll pathway and the Imd pathway, which can be triggered by the recognition of Dap-type PGN by PGRP-LCx with the assistance of PGRP-LCa in *Drosophila*. There are three isoforms of PGRP-LC including PGRP-LCx, PGRP-LCa and PGRP-LCy in *Drosophila*. Our previous study showed that a single PGRP-LC exists in bumblebees. In this present study, we prove that the bumblebee *Bombus lantschouensis* PGRP-LC (Bl-PGRP-LC) can respond to an infection with Gram-negative bacterium *Escherichia coli* through binding to the Dap-type PGNs directly, and that *E. coli* infection induces the quick and strong upregulation of *PGRP-LC*, *abaecin* and *defensin*. Moreover, the Bl-PGRP-LC exhibits a very strong affinity for the Dap-type PGN, much stronger than the affinity exhibited by the PGRP-LC from the more eusocial honeybee *Apis mellifera* (Am-PGRP-LC). In addition, mutagenesis experiments showed that the residue His^390^ is the anchor residue for the binding to the Dap-type PGN and forms a hydrogen bond with MurNAc rather than meso-Dap, which interacts with the anchor residue Arg^413^ of PGRP-LCx in *Drosophila*. Therefore, bumblebee PGRP-LC possesses exclusive characteristics for the immune response among insect PGRPs.

## 1. Introduction

Innate immunity is an important line of defense that protects insects from pathogen invasion. It can be activated by interactions between germline-encoded pattern recognition receptors (PRRs) and pathogen-associated molecular patterns (PAMPs) [1,2,3,4]. As an important type of PRR, peptidoglycan recognition proteins (PGRPs) were first discovered in the hemolymph of silkworms and participate in an antimicrobial host defense mechanism [5]. PGRPs have been shown to be conserved from mammals to insects, except in lower metazoans (nematodes) and plants that do not have PGRPs [6].

Nevertheless, insects possess more diverse PGRPs than mammals; for instance, *Drosophila melanogaster* has 13 PGRP genes and *Anopheles gambiae* possesses seven PGRP genes [6]. Based on their length, insect PGRPs can be divided into two classes: short PGRPs (PGRP-S) and long PGRPs (PGRP-L). Each PGRP contains a single peptidoglycan (PGN)-binding domain. These PGRPs recognize the bacterial or fungal PGN and then trigger two important immune pathways, the Toll pathway and the Imd pathway, which activate the production and release of antimicrobial peptides against the pathogenic infection [4,7,8]. On the basis of the components, PGNs can be classified into two major categories: L-lysine type (Lys-type) PGNs which mainly exist in Gram-positive cocci, and meso-diaminopimelic acid (Dap)-type PGNs which are present in Gram-negative bacteria and several Gram-positive bacilli [9]. The *Drosophila* PGRP-LB structure was the first for which the PGRP-binding groove was characterized [10]. Subsequently, six other *Drosophila* PGRP structures were determined, including the structures of PGRP-SA [11,12], PGRP-SD [13], PGRP-LCa [14], PGRP-LE [15], PGRP-LF [16] and the PGRP-LCx/PGRP-LCa complex with tracheal cytotoxin (TCT), a naturally occurring fragment of the Gram-negative PGN [17]. Notably, the PGRP-LCx/PGRP-LCa/TCT complex structure was the first to show details of the interaction between PGRP-LC and Dap-type PGNs at the atomic level. The complex structure shows that Arg^413^ creates a positively charged patch, which is consistent with the preferential binding of PGRP-LCx to a Dap-type PGN. The residues Trp^394^, Asp^395^, His^365^, Glu^480^, Ser^477^, Ala^478^ and Thr^479^ were also shown to be essential for the ability to bind to Dap-type PGNs. *Drosophila* PGRP-LCx requires the cooperation of PGRP-LCa to bind to Dap-type PGNs to activate the Imd immune pathway [17]. Other studies have shown that *Drosophila* PGRP-LC binds to Dap-type PGNs in cooperation with PGRP-LE and triggers the Imd pathway to activate the antimicrobial peptide production and release [18].

As a group of important pollinators, bumblebees contribute greatly to the pollination of wild flowers and crops and play a key role in the maintenance of natural and agricultural ecosystems [19,20,21,22,23,24]. However, bumblebee colonies are threatened by various pathogens, which have coevolved with their immune systems for many years [25,26,27,28,29,30]. Bumblebees belong to the Hymenoptera and are members of a more ancient clade than that of *Drosophila* (Diptera) [31]. In addition, bumblebees possess a relatively low number of immune-related genes including PGRPs, which are involved in the Toll pathway and the Imd pathway [32]. Bumblebee and honeybee PGRP-S genes have been shown to be upregulated under challenge with bacteria or the antigenic components of bacteria [33,34]. In contrast to *Drosophila* PGRP-SA, the PGRP-SA proteins of the bumblebee *Bombus ignitus* and the honeybee *Apis mellifera* preferentially bind to Dap-type PGNs over Lys-type PGNs and can bind to Dap-type PGNs with high affinity [35,36]. Interestingly, unlike *Drosophila* PGRP-LC, which has three isoforms, namely, PGRP-LCa, PGRP-LCx and PGRP-LCy, the bumblebee *B*. *lantschouensis* PGRP-LC exists as only one isoform, which is highly conserved among different bumblebee species [37]. Nevertheless, the structural and functional details of the PGN-binding activity of PGRP-LC in bees remain unclear, impeding our understanding of the immune role of PGRP-LC in the pathogen defense of bees. In this current study, the structural and functional characteristics of *B. lantschouensis* PGRP-LC were investigated to fill this knowledge gap regarding PGRP-LC in bees.

## 2. Results

### 2.1. Response of Bl-PGPR-LC to Infection with Gram-Negative and Gram-Positive Bacteria

**Figure 1:**
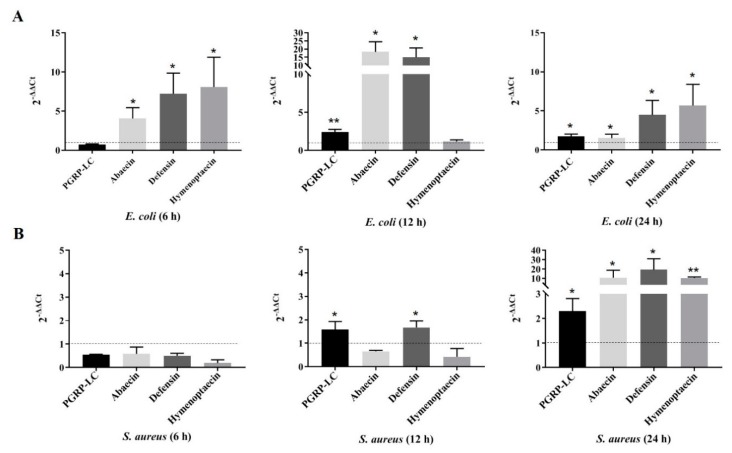
Changes in the expression levels of the *PGRP-LC*, *abaecin*, *defensin* and *hymenoptaecin* genes in the bumblebee *Bombus lantschouensis* after challenge with the Gram-negative bacterium *E. coli* and the Gram-positive bacterium *S. aureus*. (**A**) Changes in the expression levels of the *PGRP-LC*, *abaecin*, *defensin* and *hymenoptaecin* genes induced by the infection with *E. coli* at 6 h, 12 h and 24 h in *B. lantschouensis* workers. (**B**) Changes in the expression levels of the *PGRP-LC*, *abaecin*, *defensin* and *hymenoptaecin* genes in *B. lantschouensis* workers triggered by the *S. aureus* infection at 6 h, 12 h and 24 h. The X-axis indicates the gene names, and the Y-axis indicates the 2^−^^△△Ct^ value. ** indicates *p*-value < 0.01, and * indicates *p*-value between 0.01 and 0.05. The dashed line indicates the differential expression level of the four genes in the control group (a 2^−^^ΔΔCt^ value of 1).

The present study showed that both the Gram-positive bacterium *Staphylococcus aureus* and the Gram-negative bacterium *Escherichia coli* can trigger the upregulation of *PGRP-LC* and the antimicrobial peptides *abaecin*, *defensin* and *hymenoptaecin* (Figure 1). However, over time, the degree of response differs between *S. aureus* and *E. coli.* After *E. coli* infection, the *PGRP-LC* gene showed no change within 6 h, while *hymenoptaecin* was significantly upregulated (Figure 1A). After 12 h, the *PGRP-LC*, *abaecin* and *defensin* genes were strongly upregulated (Figure 1A). However, after 24 h, an obvious decrease in the degree of upregulation of the *PGRP-LC*, *abaecin* and *defensin* genes was observed compared to that at 12 h of infection; in contrast, *hymenoptaecin* was upregulated, which was similar to the result at 6 h of infection (Figure 1A). In contrast to the *E. coli* infection, the *S. aureus* infection only caused the upregulation of *PGRP-LC* and *defensin* after 12 h and none of the four genes were upregulated within 6 h (Figure 1B). After 24 h, all four genes were upregulated significantly, and the upregulation was obviously higher than that at 12 h of infection (Figure 1B).

### 2.2. Bombus PGRP-LC Strongly Bound to Dap-Type PGN and Exhibited Unique Anchor Residues in Comparison with Drosophila PGRP-LCx

**Figure 2:**
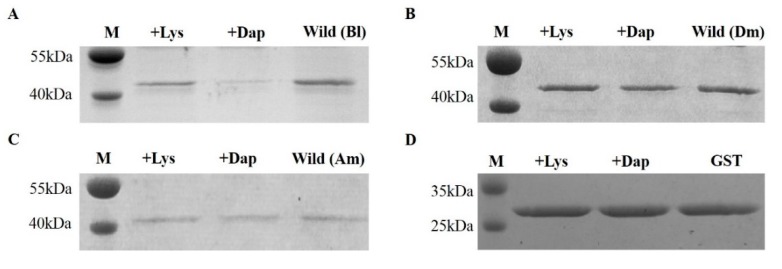
Analysis of the peptidoglycan (PGN)-binding ability of the wild-type bumblebee *Bombus lantschouensis* PGRP-LC (Bl-PGRP-LC) and honeybee *Apis mellifera* PGRP-LC (Am-PGRP-LC). Each sample includes four lanes. The first lane (M) is the marker; the second lane (+Lys) shows the corresponding protein samples after pull-down with the meso-diaminopimelic acid (Dap-type) PGN from *B. subtilis*; the third lane (+Dap) shows the corresponding protein samples after pull-down with the L-lysine type (Lys-type) PGN from *M. luteus*; and the fourth lane shows the normal corresponding protein as a control. (**A**) Analysis of the PGN-binding ability of the wild-type Bl-PGRP-LC. (**B**) The analysis of the PGN-binding ability of the wild-type Dm-PGRP-LC. (**C**) Analysis of the PGN-binding ability of the wild-type Am-PGRP-LC. (**D**) Analysis of the PGN-binding ability of the GST-tagged protein.

**Figure 3:**
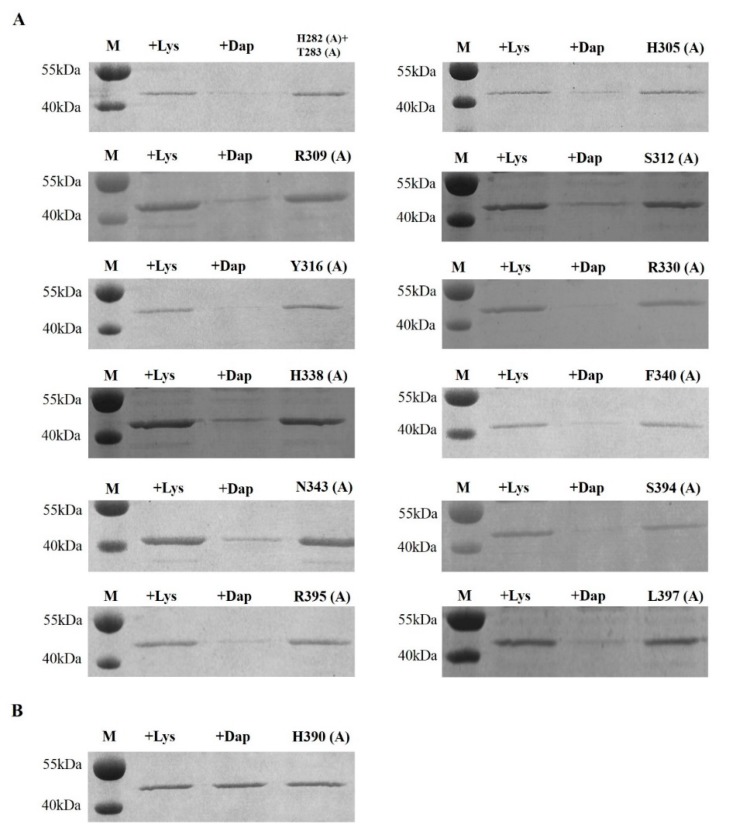
Analysis of the PGN-binding ability of the bumblebee *Bombus lantschouensis* PGRP-LC (Bl-PGRP-LC) mutants. Each sample includes four lanes. The first lane (M) is the marker; the second lane (+Lys) shows the corresponding protein samples after pull-down with the Dap-type PGN from *B. subtilis*; the third lane (+Dap) shows the corresponding protein samples after pull-down with the Lys-type PGN from *M. luteus*; and the fourth lane shows the Bl-PGRP-LC mutant protein. (**A**) Analysis of the PGN-binding ability of the twelve Bl-PGRP-LC mutants, namely, H283A and T283A, H305A, R309A, S312A, Y316A, R330A, H338A, F340A, N343A, S394A, R395A and L397A. (**B**) Analysis of the PGN-binding ability of the Bl-PGRP-LC mutant H390A.

The bumblebee *Bl-PGRP-LC* is upregulated after infection with *S. aureus* and *E. coli*, so the bumblebee Bl-PGRP-LC protein should have the ability to bind to the Lys-type PGN and the Dap-type PGN. However, the extracellular region of Bl-PGRP-LC is able to bind to the Dap-type PGN rather than the Lys-type PGN and shows a high affinity for the Dap-type PGN (Figure 2A). As a control, the known Dm-PGRP-LCx exhibited no binding to the Lys-type PGN or the Dap-type PGN (Figure 2B). Interestingly, the honeybee (*A. mellifera*) Am-PGRP-LC exhibited a weak affinity for the Dap-type PGN and no binding to the Lys-type PGN, but the short PGRP-LC had no ability to bind to the Dap-type PGN or the Lys-type PGN (Figure 2C). To eliminate the influence of the Glutathione S-transferase (GST) tag on the binding of PGNs, the GST protein was purified and the binding test was performed on the PGNs. The GST protein did not bind to the Dap-type PGN or the Lys-type PGN (Figure 2D). Based on the contact residues of *Drosophila* PGRP-LCx for the binding of TCT from the PGRP-LCx/PGRP-LCa/TCT complex, we mutated the 14 corresponding residues of Bl-PGRP-LC, namely, His^282^, Thr^283^, His^305^, Arg^309^, Ser^312^, Tyr^316^, Arg^330^, His^338^, Phe^340^, Asn^343^, His^390^, Ser^394^, Arg^395^ and Leu^397^. All these residues were mutated to Ala. None of these mutations showed any effect on the recognition of Lys-type PGN by Bl-PGRP-LC (Figure 3). In addition, the His^282^, Thr^283^, His^305^, Arg^309^, Ser^312^, Tyr^316^, Arg^330^, His^338^, Phe^340^, Asn^343^, Ser^394^, Arg^395^ and Leu^397^ mutations did not alter the binding to Dap-type PGN (Figure 3A). Remarkably, the mutation of the His^390^ of bumblebee Bl-PGRP-LC completely abolished binding to the Dap-type PGN (Figure 3B). Therefore, we hypothesize that the residue His^390^ is the main anchor residue of Bl-PGRP-LC for the recognition of the Dap-type PGN.

### 2.3. Structural Insight into PGRP-LC from Different Bee Species

**Figure 4:**
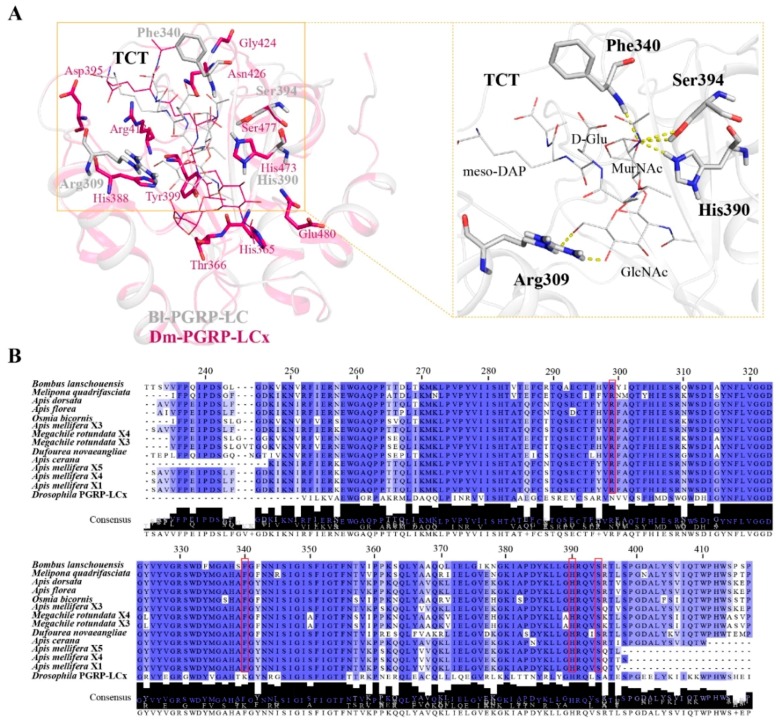
Analysis of the bumblebee Bl-PGRP-LC structure and the sequence alignment of PGRP-LC from bees. (**A**) Comparison of the Bl-PGRP-LC/TCT complex and the Dm-PGRP-LCx/TCT complex, colored in gray and magenta, respectively. TCT is shown as a line and the contact residues are shown as sticks. The yellow dashed line indicates the bonds between the TCT and the contact residues in the Bl-PGRP-LC/TCT structure. (**B**) Amino acid sequence alignment of PGRP-LC from different bee species (*Melipona quadrifasciata* GenBank No. KOX71517.1, *Apis dorsata* GenBank No. XP_006618726.1, *Apis florea* GenBank No. XP_003693124.1, *Osmia bicornis* GenBank No. XP_029039739.1, *Apis mellifera* X3 GenBank No. XP_006565566.1, *Megachile rotundata* X4 GenBank No. XP_003702406.2, *Megachile rotundata* X3 GenBank No. XP_012138567.1, *Dufourea novaeangliae* GenBank No. KZC08445.1, *Apis cerana* GenBank No. XP_AGM19450.1, *Apis mellifera* X5 GenBank No. XP_006565568.1, *Apis mellifera* X4 GenBank No. XP_006565567.1, *Apis mellifera* X3 GenBank No. XP_392452.2 and *Drosophila* PGRP-LCx GenBank No. 2f2l_X). The residues of Bl-PGRP-LC contributing to the TCT binding are boxed with red rectangles.

The overall structure of Bl-PGRP-LC retains the classical PGRP structure and overlaps with *Drosophila* PGRP-LCx. The residues His^365^, Thr^366^, His^388^, Asp^395^, Tyr^399^, Arg^413^, Gly^424^, Asn^426^, His^473^, Ser^477^ and Glu^480^ of *Drosophila* Dm-PGRP-LCx respond to the binding to the Dap-type PGN. In comparison, the Bl-PGRP-LC/TCT complex structure shows that the residues Arg^309^, Phe^340^, His^390^ (His^473^ in Dm-PGRP-LCx) and Ser^394^ (Ser^477^ in Dm-PGRP-LCx) interact with TCT and stabilize the binding of TCT (Figure 4A). We found that His^390^ is the anchor amino acid for *Bombus* Bl-PGRP-LC, which is different from the anchor amino acid Arg^413^ in *Drosophila* Dm-PGPR-LCx (Figure 3B). Moreover, the residue His^390^ forms a hydrogen bond with the MurNAc of TCT rather than the meso-Dap of TCT, which interacts with the corresponding anchor residue Arg^413^ in Dm-PGRP-LCx. Therefore, we hypothesize that the residues that stabilize the interaction between Bl-PGRP-LC and the Dap-type PGN are not conserved in *Drosophila* Dm-PGRP-LCx, especially the anchor residue, and that the binding to the Dap-type PGN might be different. Moreover, the extracellular regions of PGRP-LCs of different bee species, including *Apis cerana*, *A. mellifera*, *A. dorsata*, *A. florea*, *Bombus lantschouensis*, *Dufourea novaeangliae*, *Melipona quadrifasciata*, *Osmia bicornis* and *Megachile rotundata*, share a high sequence identity. The anchor amino acid His^390^ and the other contact residues Arg^309^, Phe^340^ and Ser^394^ are completely conserved among the different bee species (Figure 4B). Hence, we hypothesize that the structural characteristics of PGRP-LC for Dap-type PGN binding are extremely similar among bees.

## 3. Discussion

The bumblebee has fewer genes for innate immunity, which is the unique line of defense against invading pathogens, than the dipteran insects *D. melanogaster* and *A. gambiae*. These few immune genes are involved in the recognition, signaling and effector functions, such as genes encoding PGRPs, which are important PRRs and can recognize PGNs to activate important immune pathways, the Toll and Imd pathways [2,6,31,32,34]. In contrast to the corresponding *Drosophila* protein, our recent study proved that bumblebee PGRP-SA preferentially binds to the Dap-type PGN rather than the Lys-PGN, and that the residue that responds to the binding to the Dap-type PGN is conserved in Apidae [35]. Our subsequent study proved that the bumblebee *B. lantschouensis* PGRP-LC exists as a single isoform, notably different from the three isoforms observed in *Drosophila* [37]. In this study, *B. lantschouensis* PGRP-LC was shown to be able to respond to the Gram-negative bacterium *E. coli*, and Bl-PGRP-LC was able to bind to the DAP-type PGN directly. *E. coli* infection triggers a quicker and stronger upregulation of genes, including *PGRP-LC* and the antimicrobial peptide genes *abaecin* and *defensin,* than an infection with *S. aureus*. *PGRP-LC* expression showed no change at 6 h, and the reaction of *PGRP-LC* peaked at 12 h after *E. coli* infection. Although the upregulation level of *PGRP-LC* was high at 24 h after *S. aureus* infection, Bl-PGRP-LC could not bind to the Lys-type PGN, so this upregulation might be mediated by another unknown mechanism rather than by the recognition of the Lys-type PGN by PGRP-LC. Therefore, we hypothesize that the cell wall of Gram-negative bacteria first needs to be degraded to be recognized by PGRP-LC after infection in bumblebees. These results also confirm that PGRP-LC recognizes Dap-type PGNs that constitute the bacterial cell wall [6,9]. Moreover, bumblebee PGRP-LC preferentially responded to Gram-negative bacteria through the recognition of DAP-type PGNs, where PGRP-LC expression in infected bumblebees showed a parabolic trend between 6 h and 24 h and reached a maximum at 12 h.

Moreover, bumblebee Bl-PGRP-LC could recognize Dap-type PGNs with a very high affinity, and this result was consistent with the observation that bumblebee PGRP-LC exhibited an immune response to Gram-negative bacteria. However, *Drosophila* PGRP-LCx has been reported to recognize Dap-type PGNs with the cooperation of PGRP-LCa, and the *Drosophila* PGRP-LCa/PGRP-LCx/TCT complex determines the contact residues contributing to the interactions between the TCT and PGRP-LCx, especially the anchor residue Arg^413^, which interacts with meso-Dap [17]. Nevertheless, the mutation of the 12 corresponding residues did not affect the binding to the Dap-type PGN, except for the His^390^Ala mutation, which completely abolished the binding to the Dap-type PGN in Bl-PGRP-LC (Figure 3). The corresponding residue His^473^ in *Drosophila* PGRP-LCx only participated in the interactions; hence, we proved that Bl-PGRP-LC adopts the distinct anchor residue His^390^ to form hydrogen bonds with MurNAc rather than meso-Dap to stabilize the interaction with the Dap-type PGN. The Bl-PGRP-LC/TCT complex showed that the help of residues Arg^309^, Phe^340^ and Ser^394^ is required for stabilization. For now, we confirm that bumblebee PGRP-LC exhibits specific binding characteristics for the Dap-type PGN, and that these contact residues are conserved among different bee species. The results indicate that although bumblebees possess relatively few PGRPs, these limited PGRPs have evolved a precise function and structure for the recognition of diverse pathogens.

The honeybee *A. mellifera* is a more eusocial insect than the bumblebee *B. lantschouensis* in Apidae [38]. *A. mellifera* possesses a special form of immunity, namely, “social immunity”; however, *B. lantschouensis* inhabits much smaller colonies than *A. mellifera*, with fewer social divisions, which might greatly diminish the defense provided by “social immunity” [39,40]. Therefore, the higher affinity of Bl-PGRP-LC for the Dap-type PGN than that of Am-PGRP-LC might reflect a stronger individual antibacterial immunity in bumblebees than in honeybees. Moreover, bumblebee PGRP-SA also showed a higher affinity for the Dap-type PGN than that of honeybee PGRP-SA in a previous study [35]. However, more studies on the mechanism of activation of the Toll and Imd pathways by PGRPs are needed, and would help explain the specific immune characteristics of bees and the interesting differences in the immune responses of different bee species.

In conclusion, bumblebee PGRP-LC could respond to an infection by Gram-negative bacteria and act as an effective receptor for binding to the Dap-type PGN. Moreover, the recognition of the Dap-type PGN is controlled by the specific anchor residue His^390^ in the immune response to bacterial infection. In addition, bumblebee Bl-PGRP-LC exhibits a stronger binding to the Dap-type PGN than honeybee Am-PGRP-LC.

## 4. Materials and Methods

### 4.1. Bacterial Infection and Real-Time Quantitative PCR Verification

Worker bees at 10 days of age with individual weights between 0.19 and 0.21 g were selected from colonies of the Asian bumblebee B. lantschouensis and then reared in separate hives in the same surroundings and fed pollen and sugars to remove unhealthy bees over the next three days. In total, 30 healthy workers (15 workers per group) were starved for 12 h in preparation for feeding with the bacteria *Staphylococcus aureus* and *Escherichia coli*. Finally, 40 µL of bacterial culture (OD value = 1.0) mixed with sugar water at 1:1 was fed to the bees, and the time of feeding for each bee was recorded. Fifteen healthy workers were used as a control, and were fed with 40 µL sugar water. After 6 h, 12 h or 24 h, workers were directly frozen in liquid nitrogen and stored at −80 °C until RNA extraction. There were 5 workers per timepoint in each treatment and control group. The total RNA of each worker from each treatment and timepoint was extracted by using TRIzol (Thermo Fisher Scientific, Waltham, MA, USA), and cDNA was synthesized with 5× All-In-One RT MasterMix with the AccuRT Genomic DNA Removal reactions (Applied Biological Materials, British Columbia, Canada). All the samples for relative quantitative real-time PCR (qRT-PCR) samples were run on an ABI 7500 system (Applied Biosystems, Thermo Fisher Scientific, Waltham, MA, USA) with Bestar SybrGreen qPCR Mastermix (DBI^®^ Bioscience, Ludwigshafen, Germany). Each reaction was performed in a total volume of 20 μL that contained 5 μL of first-strand cDNA as a template and processed with a preincubation program of 5 min at 95 °C, followed by 40 cycles of 15 s at 95 °C and 45 s at 60 °C and a final step for 10 min at 72 °C. Gene-specific primers were designed with Primer Premier 5 (PREMIER Biosoft, San Francisco, CA, USA) (Table 1). The bumblebee α-actin gene was used as an internal reference for gene expression. Gene expression levels were calculated using the 2^−ΔΔCt^ method [41]. The credibility of 2^−ΔΔCt^ values was evaluated by the *p*-value from t-test statistics. The mean threshold cycle values for each gene were obtained from three independent PCRs of the different individuals.

### 4.2. Preparation of Proteins

The wild-type *B. lantschouensis* PGRP-LC protein (Bl-PGRP-LC) was expressed and purified as described in a recent report from our laboratory [37]. *Drosophila* PGRP-LCx (Dm-PGRP-LCx) was synthesized by GENEWIZ (Suzhou, China) based on the sequence deposited in GenBank (accession number: 2f2l), *A. mellifera* PGRP-LC and all Bl-PGRP-LC mutants were obtained via PCR with primers designed by Primer Premier 5, and the three segments were ligated to the PGEX-6p-1 vector (previously stored in our laboratory). Then, these plasmids were transformed into the *E. coli* strain BL21 (DE3) for expression and purification following the protocols for the wild-type PGRP-LC. Expression from these plasmids was induced with 0.5 mM IPTG. The bacteria were harvested by centrifugation at 8000× *g* for 5 min and were then resuspended in cold phosphate-buffered saline (PBS). After sonication, the samples were centrifuged at 11,000 ×g, and the supernatants were filtered and loaded onto a GST-tag column or a His-tag column. Finally, the collected proteins were further purified by chromatography on a Superdex 200 16/60 column (GE Healthcare, Boston, MA, USA).

### 4.3. Lys-Type and Dap-Type PGN-Binding Assays

This assay was performed as previously described with minor modifications: the experiment was conducted at 4 °C by incubating 30 μg of purified wild-type Bl-PGRP-LC or mutant Bl-PGRP-LC with 300 μg of insoluble PGN in 300 μL of binding buffer containing 20 mM Tris-HCl (pH 8.0) and 50 mM NaCl on a shaking platform for 1.5 h with Dm-PGRP-LCx as a control [17]. Lys-type and Dap-type PGNs from *Micrococcus luteus* and *Bacillus subtilis* were provided by Sigma-Aldrich (Merck KGaA, Darmstadt, Germany). After centrifuging the incubation mixture at 16,000× *g* for 5 min, the bound protein was retained in the insoluble PGN pellet and the remainder of the protein was left in the supernatant. Then, 30 μL of the supernatant was used for SDS-PAGE analysis and the PGN-free Bl-PGRP-LC was visualized by staining with Coomassie blue.

### 4.4. Amino Acid Sequence Alignment and Phylogenetic and Structural Analyses

Alignment of the amino acid sequences of the PGRP-LC proteins from different bee species was performed by using the Clustal Omega server (https://www.ebi.ac.uk/Tools/msa/clustalo/) (Conway Institute UCD, Dublin, Ireland) and Jalview Desktop (http://www.jalview.org/) (University of Dundee, Dundee, UK). The structure of Bl-PGRP-LC was modeled by SWISS-MODEL (https://swissmodel.expasy.org/) (Swiss Institute of Bioinformatics Biozentrum, University of Basel Klingelbergstrasse, Basel, Switzerland) and evaluated based on the GMEQ and QMEAN values [42,43,44,45] with the template (PDB ID: 2f2l), and then, the docking of the Bl-PGRP-SA/TCT complexes was completed by the AutoDock4.2 program (The Scripps Research Institute, San Diego, CA, USA) [46]. The structural analyses were completed by the PyMOL 1.8.6 (DeLano Scientific LLC) (Schrödinger, New York, NY, USA) and CCP4 programs (Research Complex at Harwell (RCaH) STFC Rutherford Appleton Laboratory Harwell Science and Innovation Campus Didcot Oxon, Oxford, UK).

## Figures and Tables

**Table 1:** Primes of the genes for the real-time qPCR verification.

Genes	Primers Names	Primers Sequences
*Actin*	B-actin F4	ACCTCCCTTGAGAAGAGCTACG
	B-actin R4	TACCCAGGAAGGAAGGTTGG
*PGRP-LC*	LC F1	GCAGTCTTGCCAGTTCCTCTAC
	LC R1	GAAGCACCACATTCCCATC
*Abaecin*	Abaecin F1	ATATAATCCGCCACGACCG
	Abaecin R1	GGTTTGGTAATGGGTATGGC
*Defensin*	Defensin F2	TCTTGTCGCTCTTCTCTTTGTG
	Defensin R2	TCTTCTTTGTCTGTCAGCACG
*Hymenoptaecin*	Hymenoptaecin F1	CCCGTTCTTCGGTAACTGTG
	Hymenoptaecin R1	TCACTCCGTTTCTGTCGTAGAC

## Abbreviations

PGRPs	peptidoglycan recognition proteins
PGN	peptidoglycan

## References

[1] Takeuchi O., Akira S. (2009). Innate immunity to virus infection. Immunol. Rev..

[2] Akira S., Uematsu S., Takeuchi O. (2006). Pathogen recognition and innate immunity. Cell.

[3] Medzhitov R., Janeway C.A. (2002). Decoding the patterns of self and nonself by the innate immune system. Science.

[4] Hoffmann J.A. (2003). The immune response of *Drosoph*. Nature.

[5] Yoshida H., Kinoshita K., Ashida M. (1996). Purification of a peptidoglycan recognition protein from hemolymph of the silkworm, *Bombyx mori*. J. Biol. Chem..

[6] Dziarski R., Gupta D. (2006). The peptidoglycan recognition proteins (PGRPs). Genome biol..

[7] Choe K.M., Werner T., Stoven S., Hultmark D., Anderson K.V. (2002). Requirement for a peptidoglycan recognition protein (PGRP) in Relish activation and antibacterial immune responses in *Drosophila*. Science.

[8] Michel T., Reichhart J.M., Hoffmann J.A., Royet J. (2001). *Drosophila* Toll is activated by Gram-positive bacteria through a circulating peptidoglycan recognition protein. Nature.

[9] Schleifer K.H., Kandler O. (1972). Peptidoglycan types of bacterial cell walls and their taxonomic implications. Bacteriol. Rev..

[10] Kim M.S., Byun M., Oh B.H. (2003). Crystal structure of peptidoglycan recognition protein LB from *Drosophila melanogaster*. Nat. Immunol..

[11] Reiser J.B., Teyton L., Wilson I.A. (2004). Crystal structure of the *Drosophila* peptidoglycan recognition protein (PGRP)-SA at 1.56 A resolution. J. Mol. Biol..

[12] Chang C.I., Pili-Floury S., Herve M., Parquet C., Chelliah Y., Lemaitre B., Mengin-Lecreulx D., Deisenhofer J. (2004). A *Drosophila* pattern recognition receptor contains a peptidoglycan docking groove and unusual L,D-carboxypeptidase activity. PLoS Biol..

[13] Leone P., Bischoff V., Kellenberger C., Hetru C., Royet J., Roussel A. (2008). Crystal structure of *Drosophila* PGRP-SD suggests binding to DAP-type but not lysine-type peptidoglycan. Mol. Immunol..

[14] Chang C.I., Ihara K., Chelliah Y., Mengin-Lecreulx D., Wakatsuki S., Deisenhofer J. (2005). Structure of the ectodomain of *Drosophila* peptidoglycan-recognition protein LCa suggests a molecular mechanism for pattern recognition. Proc. Natl. Acad. Sci. USA.

[15] Lim J.H., Kim M.S., Kim H.E., Yano T., Oshima Y., Aggarwal K., Goldman W.E., Silverman N., Kurata S., Oh B.H. (2006). Structural basis for preferential recognition of diaminopimelic acid-type peptidoglycan by a subset of peptidoglycan recognition proteins. J. Biol. Chem..

[16] Basbous N., Coste F., Leone P., Vincentelli R., Royet J., Kellenberger C., Roussel A. (2011). The *Drosophila* peptidoglycan-recognition protein LF interacts with peptidoglycan-recognition protein LC to downregulate the Imd pathway. EMBO Rep..

[17] Chang C.I., Chelliah Y., Borek D., Mengin-Lecreulx D., Deisenhofer J. (2006). Structure of tracheal cytotoxin in complex with a heterodimeric pattern-recognition receptor. Science.

[18] Kaneko T., Yano T., Aggarwal K., Lim J.H., Ueda K., Oshima Y., Peach C., Erturk-Hasdemir D., Goldman W.E., Oh B.H. (2006). PGRP-LC and PGRP-LE have essential yet distinct functions in the *drosophila* immune response to monomeric DAP-type peptidoglycan. Nat. Immunol..

[19] Senapathi D., Carvalheiro L.G., Biesmeijer J.C., Dodson C.A., Evans R.L., McKerchar M., Morton R.D., Moss E.D., Roberts S.P., Kunin W.E. (2015). The impact of over 80 years of land cover changes on bee and wasp pollinator communities in England. Proc. Roy. Soc. B Biol. Sci..

[20] Parmentier L., Meeus I., Cheroutre L., Mommaerts V., Louwye S., Smagghe G. (2014). Commercial bumblebee hives to assess an anthropogenic environment for pollinator support: A case study in the region of Ghent (Belgium). Environ. Monit. Assess..

[21] Klatt B.K., Holzschuh A., Westphal C., Clough Y., Smit I., Pawelzik E., Tscharntke T. (2014). Bee pollination improves crop quality, shelf life and commercial value. Proc. Roy. Soc. B Biol. Sci..

[22] Eilers E.J., Kremen C., Smith Greenleaf S., Garber A.K., Klein A.M. (2011). Contribution of pollinator-mediated crops to nutrients in the human food supply. PLoS ONE.

[23] Velthuis H.H.W., van Doorn A. (2006). A century of advances in bumblebee domestication and the economic and environmental aspects of its commercialization for pollination. Apidologie.

[24] Morandin L.A., Laverty T.M., Kevan P.G. (2001). Bumble bee (Hymenoptera: Apidae) activity and pollination levels in commercial tomato greenhouses. J. Econ. Entomol..

[25] Kraus B., Page R.E. (1998). Parasites, pathogens, and polyandry in social insects. Am. Nat..

[26] Wang H., Meeus I., Smagghe G. (2016). Israeli acute paralysis virus associated paralysis symptoms, viral tissue distribution and Dicer-2 induction in bumblebee workers (*Bombus terrestris*). J. Gen. Virol..

[27] Parmentier L., Smagghe G., de Graaf D.C., Meeus I. (2016). Varroa destructor Macula-like virus, Lake Sinai virus and other new RNA viruses in wild bumblebee hosts (*Bombus pascuorum, Bombus lapidarius and Bombus pratorum*). J. Invertebr. Pathol..

[28] Cappelle K., Smagghe G., Dhaenens M., Meeus I. (2016). Israeli Acute Paralysis Virus Infection Leads to an Enhanced RNA Interference Response and Not Its Suppression in the Bumblebee *Bombus terrestris*. Viruses.

[29] Meeus I., de Miranda J.R., de Graaf D.C., Wackers F., Smagghe G. (2014). Effect of oral infection with Kashmir bee virus and Israeli acute paralysis virus on bumblebee (*Bombus terrestris*) reproductive success. J. Invertebr. Pathol..

[30] Fürst M., McMahon D.P., Osborne J., Paxton R., Brown M. (2014). Disease associations between honeybees and bumblebees as a threat to wild pollinators. Nature.

[31] Honeybee Genome Sequencing C. (2006). Insights into social insects from the genome of the honeybee *Apis mellifera*. Nature.

[32] Sadd B.M., Barribeau S.M., Bloch G., De Graaf D.C., Dearden P.K., Elsik C.G., Gadau J., Grimmelikhuijzen C.J.P., Hasselmann M., Lozier J.D. (2015). The genomes of two key bumblebee species with primitive eusocial organization. Genome Biol..

[33] You H., Wan H., Li J., Jin B.R. (2010). Molecular cloning and characterization of a short peptidoglycan recognition protein (PGRP-S) with antibacterial activity from the bumblebee *Bombus ignitus*. Dev. Comp. Immunol..

[34] Evans J.D., Aronstein K., Chen Y.P., Hetru C., Imler J.L., Jiang H., Kanost M., Thompson G.J., Zou Z., Hultmark D. (2006). Immune pathways and defence mechanisms in honey bees *Apis mellifera*. Insect. Mol. Biol..

[35] Liu Y.J., Zhao X.M., Huang J.X., Chen M.M., An J.D. (2019). Structural Insights into the Preferential Binding of PGRP-SAs from Bumblebees and Honeybees to Dap-Type Peptidoglycans Rather than Lys-Type Peptidoglycans. J. Immunol..

[36] Liu Y., Zhao X., Naeem M., An J. (2018). Crystal structure of peptidoglycan recognition protein SA in *Apis mellifera* (Hymenoptera: Apidae). Protein. Sci..

[37] Chen M., Ye N., Liu Y., An J. (2019). Preliminary analysis of PGRP-LC gene and structure characteristics in bumblebees. Sociobiology.

[38] Cardinal S., Danforth B.N. (2011). The antiquity and evolutionary history of social behavior in bees. PLoS ONE.

[39] Simone-Finstrom M. (2017). Social immunity and the superorganism: Behavioral defenses protecting honey bee colonies from pathogens and parasites. Bee World.

[40] Cremer S., Armitage S.A., Schmid-Hempel P. (2007). Social immunity. Curr. Biol..

[41] Livak K.J., Schmittgen T.D. (2001). Analysis of relative gene expression data using real-time quantitative PCR and the 2^−ΔΔCT^ method. Methods.

[42] Waterhouse A., Bertoni M., Bienert S., Studer G., Tauriello G., Gumienny R., Heer F.T., de Beer T.A.P., Rempfer C., Bordoli L. (2018). SWISS-MODEL: Homology modelling of protein structures and complexes. Nucleic Acids Res..

[43] Biasini M., Bienert S., Waterhouse A., Arnold K., Studer G., Schmidt T., Kiefer F., Gallo Cassarino T., Bertoni M., Bordoli L. (2014). SWISS-MODEL: Modelling protein tertiary and quaternary structure using evolutionary information. Nucleic Acids Res..

[44] Guex N., Peitsch M.C., Schwede T. (2009). Automated comparative protein structure modeling with SWISS-MODEL and Swiss-PdbViewer: A historical perspective. Electrophoresis.

[45] Bordoli L., Kiefer F., Arnold K., Benkert P., Battey J., Schwede T. (2009). Protein structure homology modeling using SWISS-MODEL workspace. Nat. Protoc..

[46] Morris G.M., Huey R., Lindstrom W., Sanner M.F., Belew R.K., Goodsell D.S., Olson A.J. (2009). AutoDock4 and AutoDockTools4: Automated docking with selective receptor flexibility. J. Comput. Chem..

